# Supplementary material for: MetaRibo-Seq measures translation in microbiomes
Source: Nat Commun. 2020 Jun 29;11:3268. doi: 10.1038/s41467-020-17081-z (PMC7324362; doi:10.1038/s41467-020-17081-z)
Supplement: Supplementary file 10 — Supplementary Data 7 [file 41467_2020_17081_MOESM10_ESM.zip › File2/Confidence_High_Taxonomy/158497_out.krona.html]

Javascript must be enabled to view this page.

members
magnitude
magnitudeUnassigned
count
unassigned
taxon
rank

158497\_out

2

2
2
superkingdom

1239
phylum
2

1
class
526524

order
1
526525

1
family
128827

species
1

SRS015217\_contig\_number\_24726
2291992

class
1
186801

186802
order
1

family
1
186806

1730
1
genus

species
1

SRS014736\_contig\_number\_6966
2058290
